# Poly[[diaqua­bis­(μ_3_-3,5-dicarb­oxy­benzo­ato-κ^3^ *O* ^1^:*O* ^3^:*O* ^5^)bis­(μ_3_-5-carb­oxy­ben­zene-1,3-dicarboxyl­ato-κ^3^ *O* ^1^:*O* ^3^:*O* ^5^)tetrakis(methylformamide-κ*O*)tri­man­ganese(II)] dimethyl­formamide tetra­solvate]

**DOI:** 10.1107/S1600536812017734

**Published:** 2012-04-28

**Authors:** Jing-Wei Lei, Cai-Xia Xie, Huai-xia Yang

**Affiliations:** a Pharmacy College, Henan University of Traditional Chinese Medicine, Zhengzhou 450008, People’s Republic of China

## Abstract

In the title complex, {[Mn_3_(C_9_H_4_O_6_)_2_(C_9_H_5_O_6_)_2_(C_3_H_7_NO)_4_(H_2_O)_2_]·4C_3_H_7_NO}_*n*_, one Mn^II^ ion sits on an inversion center, and is six-coordinated by four O atoms from four anions (monoanionic and dianionic) derived from benzene-1,3,5-tricarboxylic acid and by two dimethyl­formamide (DMF) mol­ecules in a slightly distorted octa­hedral geometry. The other Mn^II^ ion is six-coordinated by four O atoms from four monoanionic and dianionic ligands, one DMF mol­ecule and one water mol­ecule in a distorted octa­hedral geometry. The monoanionic and dianionic ligands bridge the Mn^II^ ions, resulting in the formation of a layered structure parallel to (111) in which all of the carboxyl­ate groups of the anionic ligands coordinate the Mn^II^ ions in a monodentate manner. Intra- and inter­molecular O—H⋯O hydrogen bonds are present in the structure.

## Related literature

For background information on complexes based on aromatic polycarboxyl­ate ligands see: Hu *et al.* (2011 ▶); Prajapati *et al.* (2009 ▶). 
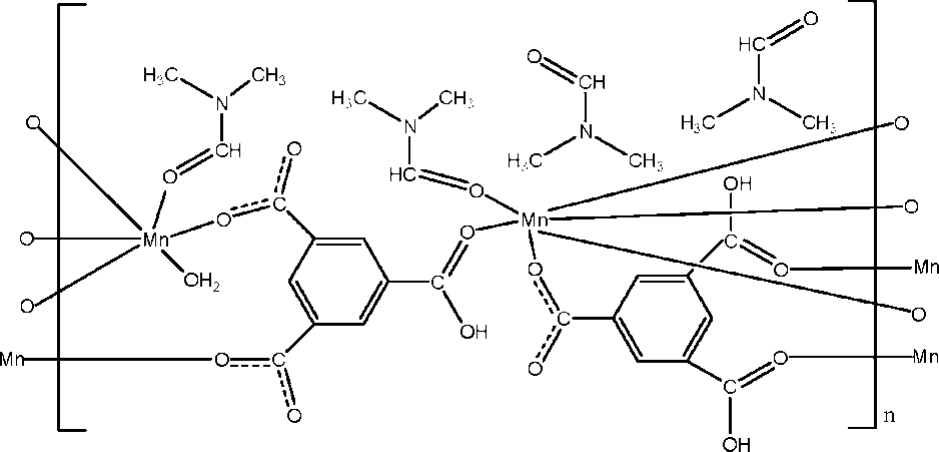


## Experimental

### 

#### Crystal data

[Mn_3_(C_9_H_4_O_6_)_2_(C_9_H_5_O_6_)_2_(C_3_H_7_NO)_4_(H_2_O)_2_]·4C_3_H_7_NO
*M* *_r_* = 1620.12
Triclinic, 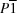

*a* = 9.826 (2) Å
*b* = 13.290 (3) Å
*c* = 14.698 (3) Å
α = 73.22 (3)°
β = 83.32 (3)°
γ = 89.79 (3)°
*V* = 1824.2 (6) Å^3^
*Z* = 1
Mo *K*α radiation
μ = 0.61 mm^−1^
*T* = 293 K
0.19 × 0.15 × 0.13 mm

#### Data collection

Rigaku Saturn CCD diffractometer
Absorption correction: multi-scan (*CrystalClear*; Rigaku/MSC, 2004 ▶) *T*_min_ = 0.894, *T*_max_ = 0.925
18985 measured reflections
6589 independent reflections
5527 reflections with *I* > 2σ(*I*)
*R*_int_ = 0.041

#### Refinement

*R*[*F* ^2^ > 2σ(*F* ^2^)] = 0.058
*wR*(*F* ^2^) = 0.116
*S* = 1.13
6589 reflections
478 parameters
H-atom parameters constrained
Δρ_max_ = 0.33 e Å^−3^
Δρ_min_ = −0.33 e Å^−3^

### 

Data collection: *CrystalClear* (Rigaku/MSC, 2004 ▶); cell refinement: *CrystalClear*; data reduction: *CrystalClear*; program(s) used to solve structure: *SHELXS97* (Sheldrick, 2008 ▶); program(s) used to refine structure: *SHELXL97* (Sheldrick, 2008 ▶); molecular graphics: *SHELXTL* (Sheldrick, 2008 ▶); software used to prepare material for publication: *publCIF* (Westrip, 2010 ▶).

## Supplementary Material

Crystal structure: contains datablock(s) global, I. DOI: 10.1107/S1600536812017734/pk2407sup1.cif

Structure factors: contains datablock(s) I. DOI: 10.1107/S1600536812017734/pk2407Isup2.hkl

Additional supplementary materials:  crystallographic information; 3D view; checkCIF report

## Figures and Tables

**Table 1:** Hydrogen-bond geometry (Å, °)

*D*—H⋯*A*	*D*—H	H⋯*A*	*D*⋯*A*	*D*—H⋯*A*
O8—H8⋯O2	0.82	1.67	2.448 (3)	157
O17—H2*W*⋯O16	0.85	1.85	2.694 (4)	176
O6—H6⋯O9^i^	0.82	1.69	2.452 (3)	153
O3—H3⋯O11^ii^	0.82	1.69	2.463 (3)	157
O17—H1*W*⋯O15^iii^	0.85	1.92	2.713 (4)	155

Symmetry codes: (i) 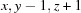; (ii) 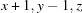; (iii) 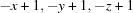.

**Fig. 1.**
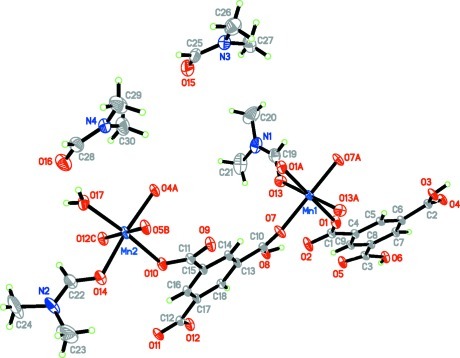
View of the title complex showing 30% probability displacement ellipsoids. [Symmetry code A: -x + 2, -y, -z + 2; B: x, y + 1, z - 1; C: -x + 1, -y + 1, -z + 2.]

**Fig. 2.**
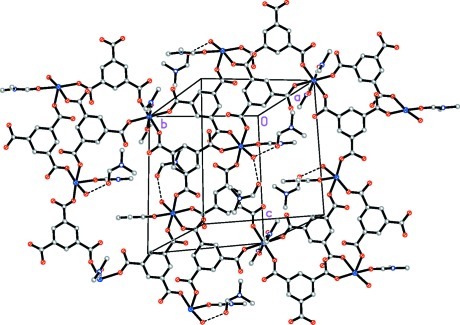
View of the two-dimensional structure of the title complex. Hydrogen atoms are omitted for clarity.

## supplementary crystallographic information

### Comment

Aromatic polycarboxylate compounds, such as 1,3,5-benzenetricarboxylic acid and 1,2,4,5-benzenetetracarboxylic acid, have been proved to be effective ligands to design and synthesize open and rigid frameworks due to the versatile binding modes and high binding capacity of the carboxylate groups (Hu *et al.*, 2011; Prajapati *et al.*, 2009). In order to further explore metal-organic frameworks with new structures, we selected 1,3,5-benzenetricarboxylic acid (H_3_btc) as ligand to self-assembly with MnCl_2_ and obtained the title complex, {[Mn_3_(C_9_H_4_O_6_)_2_(C_9_H_5_O_6_)_2_(DMF)_4_)(H_2_O)_2_](DMF)_4_}_n_, of which the crystal structure is reported herein. As shown in Figure 1, there are two crystallographically independent manganese ions (Mn1 and Mn2), two crystallographically independent 1,3,5-benzenetricarboxylte groups (Hbtc and H_2_btc), two crystallographically independent coordination DMF molecules, one coordination H_2_O molecule, two crystallographically independent uncoordination DMF molecules in an asymmetric unit. Each Mn1 ion lies on an inversion center and displays a slightly distorted octahedral geometry defined by atoms O1, O1A, O7, O7A from four 1,3,5-benzenetricarboxylte anions in equatorial positions and by atoms O13, O13A from two DMF molecules in axial positions. The Mn2 ion is bound with six oxygen atoms from four 1,3,5-benzenetricarboxylte groups (O4A, O5B, O10, O12C), one DMF molecule (O14) and one H_2_O molecule (O17) leading to a distorted octahedral geometry. As depicted in Figure 2, Mn1 and Mn2 ions are bridged by 1,3,5-benzenetricarboxylte ligands forming the two-dimensional layer structure in which all of the carboxylate groups of the 1,3,5-benzenetricarboxylte ligands coordinate to Mn^II^ ions in monodentate mode. In addition, intramolecular O—H···O hydrogen bonds between the carboxyl/carboxylate groups stabilize the molecular configuration whereas O—H···O hydrogen bonds between coordinated water molecules and solvent DMF molecules and between carboxyl/carboxylate groups of adjacent molecules consolidate the crystal packing.

### Experimental

A mixture of MnCl_2_ (0.1 mmol), 1,3,5-benzenetricarboxylic acid (0.1 mmol), *N*,*N*'-dimethylformamide (2 ml) and water (8 ml) was placed in a 25 ml Teflon-lined stainless steel vessel and heated at 100 °C for 72 h, then cooled to room temperature. Pale yellow crystals were obtained from the filtrate and dried in air.

### Refinement

H atoms bound to C atoms were positioned geometrically and refined as riding atoms, with C-H = 0.93 (aromatic, CHO) Å and 0.96 (CH_3_) Å. H atoms bound to O atoms were found in difference maps. Hydroxyl hydrogens were included using a riding model with distance restraints of O-H = 0.82 Å. Water hydrogens were included with O-H = 0.85 Å. All H atoms were assigned U_iso_(H) = 1.2 U_eq_(C,O).

### Crystal data

**Table:**

[Mn_3_(C_9_H_4_O_6_)_2_(C_9_H_5_O_6_)_2_(C_3_H_7_NO)_4_(H_2_O)_2_]·4C_3_H_7_NO	*Z* = 1
*M**_r_* = 1620.12	*F*(000) = 841
Triclinic, *P*1	*D*_x_ = 1.475 Mg m^−^^3^
*a* = 9.826 (2) Å	Mo *K*α radiation, λ = 0.71073 Å
*b* = 13.290 (3) Å	Cell parameters from 4578 reflections
*c* = 14.698 (3) Å	θ = 2.1–27.9°
α = 73.22 (3)°	µ = 0.61 mm^−^^1^
β = 83.32 (3)°	*T* = 293 K
γ = 89.79 (3)°	Prism, pale yellow
*V* = 1824.2 (6) Å^3^	0.19 × 0.15 × 0.13 mm

### Data collection

**Table:**

Rigaku Saturn CCD diffractometer	6589 independent reflections
Radiation source: fine-focus sealed tube	5527 reflections with *I* > 2σ(*I*)
Graphite monochromator	*R*_int_ = 0.041
Detector resolution: 28.6 pixels mm^-1^	θ_max_ = 25.3°, θ_min_ = 1.5°
ω scans	*h* = −11→11
Absorption correction: multi-scan (*CrystalClear*; Rigaku/MSC, 2004)	*k* = −15→15
*T*_min_ = 0.894, *T*_max_ = 0.925	*l* = −17→17
18985 measured reflections	

### Refinement

**Table:**

Refinement on *F*^2^	Primary atom site location: structure-invariant direct methods
Least-squares matrix: full	Secondary atom site location: difference Fourier map
*R*[*F*^2^ > 2σ(*F*^2^)] = 0.058	Hydrogen site location: inferred from neighbouring sites
*wR*(*F*^2^) = 0.116	H-atom parameters constrained
*S* = 1.13	*w* = 1/[σ^2^(*F*_o_^2^) + (0.0375*P*)^2^ + 1.4521*P*] where *P* = (*F*_o_^2^ + 2*F*_c_^2^)/3
6589 reflections	(Δ/σ)_max_ = 0.001
478 parameters	Δρ_max_ = 0.33 e Å^−^^3^
0 restraints	Δρ_min_ = −0.33 e Å^−^^3^

### Special details

**Table:**

Geometry. All e.s.d.'s (except the e.s.d. in the dihedral angle between two l.s. planes) are estimated using the full covariance matrix. The cell e.s.d.'s are taken into account individually in the estimation of e.s.d.'s in distances, angles and torsion angles; correlations between e.s.d.'s in cell parameters are only used when they are defined by crystal symmetry. An approximate (isotropic) treatment of cell e.s.d.'s is used for estimating e.s.d.'s involving l.s. planes.
Refinement. Refinement of *F*^2^ against ALL reflections. The weighted *R*-factor *wR* and goodness of fit *S* are based on *F*^2^, conventional *R*-factors *R* are based on *F*, with *F* set to zero for negative *F*^2^. The threshold expression of *F*^2^ > σ(*F*^2^) is used only for calculating *R*-factors(gt) *etc*. and is not relevant to the choice of reflections for refinement. *R*-factors based on *F*^2^ are statistically about twice as large as those based on *F*, and *R*- factors based on ALL data will be even larger.

### Fractional atomic coordinates and isotropic or equivalent isotropic displacement parameters (Å^2^)

**Table:**

	*x*	*y*	*z*	*U*_iso_*/*U*_eq_	
Mn1	1.0000	0.0000	1.0000	0.02695 (18)	
Mn2	0.64081 (5)	0.63958 (4)	0.66415 (3)	0.02946 (15)	
O1	1.0590 (2)	−0.06181 (19)	1.14191 (14)	0.0397 (6)	
O2	0.8883 (3)	−0.04824 (19)	1.25050 (16)	0.0455 (7)	
O3	1.3739 (2)	−0.35363 (17)	1.21994 (15)	0.0367 (6)	
H3	1.4278	−0.3959	1.2067	0.044*	
O4	1.3256 (2)	−0.48344 (17)	1.35625 (15)	0.0347 (6)	
O5	0.8257 (2)	−0.31041 (18)	1.56693 (15)	0.0368 (6)	
O6	0.9930 (2)	−0.42307 (18)	1.60883 (14)	0.0366 (6)	
H6	0.9436	−0.4415	1.6601	0.044*	
O7	0.9379 (2)	0.14743 (17)	1.02342 (16)	0.0399 (6)	
O8	0.7940 (3)	0.11542 (18)	1.15860 (16)	0.0433 (6)	
H8	0.8405	0.0635	1.1760	0.052*	
O9	0.9107 (2)	0.49197 (19)	0.77771 (15)	0.0404 (6)	
O10	0.7326 (2)	0.59048 (17)	0.79841 (14)	0.0352 (6)	
O11	0.5417 (2)	0.55479 (17)	1.13690 (15)	0.0361 (6)	
O12	0.5501 (2)	0.39777 (18)	1.24445 (15)	0.0371 (6)	
O13	0.7895 (3)	−0.0676 (2)	1.03997 (18)	0.0452 (6)	
O14	0.6417 (3)	0.8009 (2)	0.67832 (19)	0.0494 (7)	
O15	0.7270 (3)	0.2236 (3)	0.4303 (3)	0.0780 (10)	
O16	0.5669 (4)	0.7479 (2)	0.3566 (2)	0.0724 (10)	
O17	0.5163 (3)	0.6835 (2)	0.54908 (16)	0.0487 (7)	
H1W	0.4309	0.6935	0.5578	0.058*	
H2W	0.5356	0.7017	0.4886	0.058*	
N1	0.6125 (4)	−0.1482 (3)	0.9988 (3)	0.0671 (11)	
N2	0.5441 (4)	0.9589 (3)	0.6541 (3)	0.0635 (11)	
N3	0.9217 (3)	0.1958 (3)	0.3439 (3)	0.0556 (9)	
N4	0.7209 (3)	0.6531 (3)	0.2913 (2)	0.0518 (9)	
C1	0.9950 (3)	−0.0916 (2)	1.2233 (2)	0.0286 (7)	
C2	1.3077 (3)	−0.3950 (2)	1.3036 (2)	0.0280 (7)	
C3	0.9364 (3)	−0.3492 (2)	1.5497 (2)	0.0278 (7)	
C4	1.0434 (3)	−0.1846 (2)	1.2955 (2)	0.0248 (7)	
C5	1.1553 (3)	−0.2386 (2)	1.2693 (2)	0.0251 (7)	
H5A	1.2024	−0.2142	1.2081	0.030*	
C6	1.1979 (3)	−0.3284 (2)	1.3335 (2)	0.0249 (7)	
C7	1.1277 (3)	−0.3631 (2)	1.4253 (2)	0.0267 (7)	
H7A	1.1563	−0.4225	1.4692	0.032*	
C8	1.0154 (3)	−0.3099 (2)	1.4523 (2)	0.0248 (7)	
C9	0.9725 (3)	−0.2219 (2)	1.3868 (2)	0.0256 (7)	
H9A	0.8956	−0.1874	1.4039	0.031*	
C10	0.8490 (3)	0.1743 (2)	1.0777 (2)	0.0277 (7)	
C11	0.8095 (3)	0.5173 (2)	0.8280 (2)	0.0294 (7)	
C12	0.5792 (3)	0.4611 (2)	1.1633 (2)	0.0273 (7)	
C13	0.7994 (3)	0.2843 (2)	1.0503 (2)	0.0255 (7)	
C14	0.8347 (3)	0.3501 (2)	0.9576 (2)	0.0263 (7)	
H14A	0.8927	0.3267	0.9139	0.032*	
C15	0.7838 (3)	0.4499 (2)	0.9307 (2)	0.0261 (7)	
C16	0.7011 (3)	0.4862 (2)	0.9972 (2)	0.0274 (7)	
H16A	0.6671	0.5535	0.9789	0.033*	
C17	0.6688 (3)	0.4234 (2)	1.0903 (2)	0.0241 (7)	
C18	0.7168 (3)	0.3212 (2)	1.1157 (2)	0.0249 (7)	
H18A	0.6930	0.2774	1.1774	0.030*	
C19	0.7395 (4)	−0.1154 (3)	0.9906 (3)	0.0559 (11)	
H19A	0.7990	−0.1296	0.9421	0.067*	
C20	0.5644 (6)	−0.2077 (5)	0.9397 (5)	0.116 (2)	
H20A	0.6388	−0.2159	0.8942	0.139*	
H20B	0.4923	−0.1710	0.9064	0.139*	
H20C	0.5303	−0.2757	0.9794	0.139*	
C21	0.5122 (5)	−0.1282 (4)	1.0712 (4)	0.098 (2)	
H21A	0.5550	−0.0888	1.1061	0.117*	
H21B	0.4759	−0.1939	1.1144	0.117*	
H21C	0.4392	−0.0888	1.0412	0.117*	
C22	0.5480 (4)	0.8594 (3)	0.6588 (3)	0.0552 (11)	
H22	0.4695	0.8301	0.6456	0.066*	
C23	0.6581 (6)	1.0127 (4)	0.6741 (5)	0.114 (2)	
H23A	0.7306	0.9646	0.6903	0.137*	
H23B	0.6298	1.0402	0.7267	0.137*	
H23C	0.6903	1.0695	0.6185	0.137*	
C24	0.4228 (6)	1.0195 (5)	0.6292 (5)	0.127 (3)	
H24A	0.3539	0.9750	0.6180	0.153*	
H24B	0.4468	1.0768	0.5724	0.153*	
H24C	0.3879	1.0467	0.6809	0.153*	
C25	0.8121 (5)	0.2499 (4)	0.3601 (4)	0.0607 (12)	
H25A	0.7995	0.3129	0.3142	0.073*	
C26	1.0185 (5)	0.2309 (4)	0.2581 (3)	0.0760 (14)	
H26A	0.9883	0.2951	0.2171	0.091*	
H26B	1.0241	0.1780	0.2251	0.091*	
H26C	1.1071	0.2427	0.2752	0.091*	
C27	0.9503 (5)	0.0999 (4)	0.4130 (4)	0.0749 (14)	
H27A	0.8797	0.0847	0.4664	0.090*	
H27B	1.0372	0.1073	0.4346	0.090*	
H27C	0.9533	0.0435	0.3842	0.090*	
C28	0.6549 (5)	0.7381 (3)	0.2937 (3)	0.0561 (11)	
H28	0.6772	0.7976	0.2421	0.067*	
C29	0.8230 (5)	0.6526 (4)	0.2122 (4)	0.0839 (17)	
H29A	0.8311	0.7212	0.1666	0.101*	
H29B	0.7959	0.6020	0.1819	0.101*	
H29C	0.9097	0.6342	0.2355	0.101*	
C30	0.6934 (5)	0.5587 (4)	0.3695 (4)	0.0809 (16)	
H30A	0.6237	0.5713	0.4161	0.097*	
H30B	0.7757	0.5383	0.3986	0.097*	
H30C	0.6625	0.5034	0.3461	0.097*	

### Atomic displacement parameters (Å^2^)

**Table:**

	*U*^11^	*U*^22^	*U*^33^	*U*^12^	*U*^13^	*U*^23^
Mn1	0.0340 (4)	0.0230 (4)	0.0199 (3)	0.0078 (3)	0.0035 (3)	−0.0028 (3)
Mn2	0.0318 (3)	0.0259 (3)	0.0237 (3)	0.0107 (2)	0.0054 (2)	0.0004 (2)
O1	0.0471 (14)	0.0431 (14)	0.0181 (11)	0.0165 (12)	0.0016 (10)	0.0054 (10)
O2	0.0545 (16)	0.0367 (14)	0.0310 (13)	0.0262 (12)	0.0112 (12)	0.0059 (11)
O3	0.0433 (14)	0.0298 (13)	0.0285 (12)	0.0131 (11)	0.0158 (11)	−0.0034 (10)
O4	0.0436 (14)	0.0260 (12)	0.0286 (12)	0.0148 (11)	0.0033 (10)	−0.0020 (10)
O5	0.0352 (13)	0.0341 (13)	0.0299 (12)	0.0105 (11)	0.0121 (10)	0.0019 (10)
O6	0.0400 (14)	0.0389 (14)	0.0187 (11)	0.0138 (11)	0.0079 (10)	0.0062 (10)
O7	0.0531 (15)	0.0250 (13)	0.0356 (13)	0.0154 (11)	0.0092 (12)	−0.0051 (10)
O8	0.0528 (16)	0.0280 (13)	0.0353 (13)	0.0196 (12)	0.0081 (12)	0.0069 (11)
O9	0.0427 (14)	0.0458 (15)	0.0214 (11)	0.0187 (12)	0.0107 (10)	0.0024 (11)
O10	0.0445 (14)	0.0304 (13)	0.0230 (12)	0.0138 (11)	−0.0003 (10)	0.0028 (10)
O11	0.0420 (14)	0.0272 (13)	0.0334 (13)	0.0096 (11)	0.0120 (11)	−0.0061 (10)
O12	0.0383 (13)	0.0415 (14)	0.0226 (12)	0.0127 (11)	0.0104 (10)	−0.0009 (11)
O13	0.0425 (15)	0.0472 (16)	0.0432 (15)	−0.0054 (12)	0.0042 (12)	−0.0125 (13)
O14	0.0489 (16)	0.0382 (15)	0.0610 (17)	0.0145 (13)	−0.0058 (13)	−0.0146 (13)
O15	0.059 (2)	0.100 (3)	0.083 (2)	0.029 (2)	−0.0168 (19)	−0.037 (2)
O16	0.106 (3)	0.063 (2)	0.0425 (16)	0.0293 (19)	0.0030 (17)	−0.0113 (15)
O17	0.0462 (15)	0.0636 (18)	0.0297 (13)	0.0191 (13)	−0.0018 (11)	−0.0045 (12)
N1	0.048 (2)	0.051 (2)	0.091 (3)	−0.0097 (18)	−0.018 (2)	0.001 (2)
N2	0.063 (2)	0.0298 (18)	0.093 (3)	0.0087 (17)	0.012 (2)	−0.0193 (19)
N3	0.050 (2)	0.059 (2)	0.061 (2)	0.0103 (18)	−0.0139 (18)	−0.0199 (19)
N4	0.050 (2)	0.049 (2)	0.062 (2)	0.0134 (17)	−0.0142 (17)	−0.0202 (18)
C1	0.0355 (18)	0.0255 (17)	0.0224 (16)	0.0061 (14)	−0.0014 (14)	−0.0039 (14)
C2	0.0293 (17)	0.0297 (18)	0.0245 (16)	0.0071 (14)	0.0000 (14)	−0.0083 (14)
C3	0.0346 (18)	0.0228 (16)	0.0219 (16)	0.0029 (14)	0.0041 (14)	−0.0032 (13)
C4	0.0301 (16)	0.0206 (16)	0.0205 (15)	0.0041 (13)	0.0000 (13)	−0.0020 (13)
C5	0.0311 (17)	0.0238 (16)	0.0178 (15)	0.0033 (13)	0.0024 (13)	−0.0042 (13)
C6	0.0261 (16)	0.0250 (16)	0.0213 (15)	0.0053 (13)	0.0038 (13)	−0.0059 (13)
C7	0.0312 (17)	0.0241 (16)	0.0206 (15)	0.0079 (14)	−0.0011 (13)	−0.0006 (13)
C8	0.0284 (16)	0.0267 (17)	0.0170 (15)	0.0047 (13)	0.0026 (13)	−0.0048 (13)
C9	0.0295 (17)	0.0245 (16)	0.0211 (15)	0.0082 (13)	0.0005 (13)	−0.0055 (13)
C10	0.0335 (18)	0.0233 (17)	0.0232 (16)	0.0077 (14)	−0.0033 (14)	−0.0021 (14)
C11	0.0358 (18)	0.0261 (17)	0.0216 (16)	0.0066 (15)	0.0011 (14)	−0.0013 (14)
C12	0.0258 (16)	0.0291 (18)	0.0240 (16)	0.0019 (14)	0.0036 (13)	−0.0058 (14)
C13	0.0294 (17)	0.0233 (16)	0.0221 (16)	0.0086 (13)	−0.0040 (13)	−0.0035 (13)
C14	0.0300 (17)	0.0287 (17)	0.0184 (15)	0.0101 (14)	0.0037 (13)	−0.0065 (13)
C15	0.0305 (17)	0.0259 (17)	0.0181 (15)	0.0093 (14)	−0.0003 (13)	−0.0012 (13)
C16	0.0304 (17)	0.0215 (16)	0.0258 (16)	0.0078 (13)	−0.0006 (14)	−0.0011 (13)
C17	0.0251 (16)	0.0260 (16)	0.0190 (15)	0.0056 (13)	0.0015 (13)	−0.0050 (13)
C18	0.0296 (16)	0.0229 (16)	0.0177 (15)	0.0032 (13)	0.0022 (13)	−0.0009 (13)
C19	0.053 (3)	0.045 (2)	0.062 (3)	0.003 (2)	−0.005 (2)	−0.005 (2)
C20	0.111 (5)	0.082 (4)	0.162 (6)	−0.023 (4)	−0.067 (5)	−0.027 (4)
C21	0.057 (3)	0.081 (4)	0.114 (5)	−0.011 (3)	0.006 (3)	0.030 (3)
C22	0.050 (2)	0.043 (2)	0.075 (3)	0.008 (2)	−0.003 (2)	−0.022 (2)
C23	0.108 (5)	0.063 (4)	0.181 (7)	−0.008 (3)	−0.005 (5)	−0.055 (4)
C24	0.102 (5)	0.064 (4)	0.218 (8)	0.044 (4)	−0.017 (5)	−0.045 (5)
C25	0.057 (3)	0.058 (3)	0.077 (3)	0.016 (2)	−0.036 (3)	−0.025 (3)
C26	0.059 (3)	0.086 (4)	0.082 (4)	−0.011 (3)	−0.011 (3)	−0.022 (3)
C27	0.076 (3)	0.065 (3)	0.086 (4)	0.027 (3)	−0.018 (3)	−0.021 (3)
C28	0.074 (3)	0.050 (3)	0.041 (2)	0.013 (2)	−0.006 (2)	−0.007 (2)
C29	0.051 (3)	0.100 (4)	0.116 (4)	0.006 (3)	0.008 (3)	−0.061 (4)
C30	0.092 (4)	0.048 (3)	0.104 (4)	0.023 (3)	−0.034 (3)	−0.014 (3)

### Geometric parameters (Å, º)

**Table:**

Mn1—O1^i^	2.156 (2)	C3—C8	1.496 (4)
Mn1—O1	2.156 (2)	C4—C5	1.388 (4)
Mn1—O7^i^	2.159 (2)	C4—C9	1.390 (4)
Mn1—O7	2.159 (2)	C5—C6	1.388 (4)
Mn1—O13^i^	2.204 (3)	C5—H5A	0.9300
Mn1—O13	2.204 (3)	C6—C7	1.392 (4)
Mn2—O12^ii^	2.146 (2)	C7—C8	1.389 (4)
Mn2—O17	2.146 (3)	C7—H7A	0.9300
Mn2—O5^iii^	2.154 (2)	C8—C9	1.383 (4)
Mn2—O10	2.191 (2)	C9—H9A	0.9300
Mn2—O4^i^	2.199 (2)	C10—C13	1.496 (4)
Mn2—O14	2.213 (3)	C11—C15	1.509 (4)
O1—C1	1.239 (3)	C12—C17	1.504 (4)
O2—C1	1.274 (4)	C13—C18	1.383 (4)
O3—C2	1.290 (4)	C13—C14	1.394 (4)
O3—H3	0.8200	C14—C15	1.380 (4)
O4—C2	1.231 (4)	C14—H14A	0.9300
O4—Mn2^i^	2.199 (2)	C15—C16	1.390 (4)
O5—C3	1.233 (4)	C16—C17	1.382 (4)
O5—Mn2^iv^	2.154 (2)	C16—H16A	0.9300
O6—C3	1.283 (4)	C17—C18	1.396 (4)
O6—H6	0.8200	C18—H18A	0.9300
O7—C10	1.236 (4)	C19—H19A	0.9300
O8—C10	1.278 (4)	C20—H20A	0.9600
O8—H8	0.8200	C20—H20B	0.9600
O9—C11	1.273 (4)	C20—H20C	0.9600
O10—C11	1.238 (4)	C21—H21A	0.9600
O11—C12	1.261 (4)	C21—H21B	0.9600
O12—C12	1.247 (4)	C21—H21C	0.9600
O12—Mn2^ii^	2.146 (2)	C22—H22	0.9300
O13—C19	1.234 (5)	C23—H23A	0.9600
O14—C22	1.210 (4)	C23—H23B	0.9600
O15—C25	1.215 (5)	C23—H23C	0.9600
O16—C28	1.226 (5)	C24—H24A	0.9600
O17—H1W	0.8500	C24—H24B	0.9600
O17—H2W	0.8500	C24—H24C	0.9600
N1—C19	1.304 (5)	C25—H25A	0.9300
N1—C21	1.446 (6)	C26—H26A	0.9600
N1—C20	1.448 (6)	C26—H26B	0.9600
N2—C22	1.304 (5)	C26—H26C	0.9600
N2—C23	1.434 (6)	C27—H27A	0.9600
N2—C24	1.459 (6)	C27—H27B	0.9600
N3—C25	1.330 (5)	C27—H27C	0.9600
N3—C27	1.432 (5)	C28—H28	0.9300
N3—C26	1.448 (5)	C29—H29A	0.9600
N4—C28	1.308 (5)	C29—H29B	0.9600
N4—C30	1.438 (5)	C29—H29C	0.9600
N4—C29	1.446 (5)	C30—H30A	0.9600
C1—C4	1.496 (4)	C30—H30B	0.9600
C2—C6	1.500 (4)	C30—H30C	0.9600
			
O1^i^—Mn1—O1	180.00 (5)	O8—C10—C13	115.1 (3)
O1^i^—Mn1—O7^i^	93.27 (9)	O10—C11—O9	125.0 (3)
O1—Mn1—O7^i^	86.73 (9)	O10—C11—C15	119.5 (3)
O1^i^—Mn1—O7	86.73 (9)	O9—C11—C15	115.5 (3)
O1—Mn1—O7	93.27 (9)	O12—C12—O11	125.8 (3)
O7^i^—Mn1—O7	180.00 (11)	O12—C12—C17	117.5 (3)
O1^i^—Mn1—O13^i^	94.50 (10)	O11—C12—C17	116.7 (3)
O1—Mn1—O13^i^	85.50 (10)	C18—C13—C14	119.3 (3)
O7^i^—Mn1—O13^i^	92.31 (10)	C18—C13—C10	120.6 (3)
O7—Mn1—O13^i^	87.69 (10)	C14—C13—C10	120.1 (3)
O1^i^—Mn1—O13	85.50 (10)	C15—C14—C13	120.2 (3)
O1—Mn1—O13	94.50 (10)	C15—C14—H14A	119.9
O7^i^—Mn1—O13	87.69 (10)	C13—C14—H14A	119.9
O7—Mn1—O13	92.31 (10)	C14—C15—C16	119.8 (3)
O13^i^—Mn1—O13	180.0	C14—C15—C11	120.4 (3)
O12^ii^—Mn2—O17	85.30 (9)	C16—C15—C11	119.7 (3)
O12^ii^—Mn2—O5^iii^	175.24 (9)	C17—C16—C15	120.8 (3)
O17—Mn2—O5^iii^	91.68 (9)	C17—C16—H16A	119.6
O12^ii^—Mn2—O10	84.37 (9)	C15—C16—H16A	119.6
O17—Mn2—O10	169.63 (9)	C16—C17—C18	118.8 (3)
O5^iii^—Mn2—O10	98.57 (9)	C16—C17—C12	121.6 (3)
O12^ii^—Mn2—O4^i^	97.58 (9)	C18—C17—C12	119.6 (3)
O17—Mn2—O4^i^	91.90 (10)	C13—C18—C17	121.0 (3)
O5^iii^—Mn2—O4^i^	86.18 (9)	C13—C18—H18A	119.5
O10—Mn2—O4^i^	90.35 (9)	C17—C18—H18A	119.5
O12^ii^—Mn2—O14	90.70 (10)	O13—C19—N1	127.2 (4)
O17—Mn2—O14	91.98 (10)	O13—C19—H19A	116.4
O5^iii^—Mn2—O14	85.72 (10)	N1—C19—H19A	116.4
O10—Mn2—O14	87.24 (10)	N1—C20—H20A	109.5
O4^i^—Mn2—O14	171.11 (9)	N1—C20—H20B	109.5
C1—O1—Mn1	134.2 (2)	H20A—C20—H20B	109.5
C2—O3—H3	109.5	N1—C20—H20C	109.5
C2—O4—Mn2^i^	135.3 (2)	H20A—C20—H20C	109.5
C3—O5—Mn2^iv^	136.4 (2)	H20B—C20—H20C	109.5
C3—O6—H6	109.5	N1—C21—H21A	109.5
C10—O7—Mn1	135.3 (2)	N1—C21—H21B	109.5
C10—O8—H8	109.5	H21A—C21—H21B	109.5
C11—O10—Mn2	130.3 (2)	N1—C21—H21C	109.5
C12—O12—Mn2^ii^	131.1 (2)	H21A—C21—H21C	109.5
C19—O13—Mn1	120.7 (3)	H21B—C21—H21C	109.5
C22—O14—Mn2	122.6 (3)	O14—C22—N2	127.6 (4)
Mn2—O17—H1W	122.8	O14—C22—H22	116.2
Mn2—O17—H2W	132.6	N2—C22—H22	116.2
H1W—O17—H2W	104.2	N2—C23—H23A	109.5
C19—N1—C21	120.3 (4)	N2—C23—H23B	109.5
C19—N1—C20	123.2 (5)	H23A—C23—H23B	109.5
C21—N1—C20	116.5 (4)	N2—C23—H23C	109.5
C22—N2—C23	121.5 (4)	H23A—C23—H23C	109.5
C22—N2—C24	121.2 (4)	H23B—C23—H23C	109.5
C23—N2—C24	117.3 (4)	N2—C24—H24A	109.5
C25—N3—C27	120.5 (4)	N2—C24—H24B	109.5
C25—N3—C26	122.5 (4)	H24A—C24—H24B	109.5
C27—N3—C26	116.9 (4)	N2—C24—H24C	109.5
C28—N4—C30	119.5 (4)	H24A—C24—H24C	109.5
C28—N4—C29	121.3 (4)	H24B—C24—H24C	109.5
C30—N4—C29	119.2 (4)	O15—C25—N3	125.2 (5)
O1—C1—O2	124.2 (3)	O15—C25—H25A	117.4
O1—C1—C4	119.2 (3)	N3—C25—H25A	117.4
O2—C1—C4	116.6 (3)	N3—C26—H26A	109.5
O4—C2—O3	125.1 (3)	N3—C26—H26B	109.5
O4—C2—C6	120.1 (3)	H26A—C26—H26B	109.5
O3—C2—C6	114.8 (3)	N3—C26—H26C	109.5
O5—C3—O6	125.7 (3)	H26A—C26—H26C	109.5
O5—C3—C8	119.5 (3)	H26B—C26—H26C	109.5
O6—C3—C8	114.8 (3)	N3—C27—H27A	109.5
C5—C4—C9	119.4 (3)	N3—C27—H27B	109.5
C5—C4—C1	119.6 (3)	H27A—C27—H27B	109.5
C9—C4—C1	120.8 (3)	N3—C27—H27C	109.5
C4—C5—C6	120.8 (3)	H27A—C27—H27C	109.5
C4—C5—H5A	119.6	H27B—C27—H27C	109.5
C6—C5—H5A	119.6	O16—C28—N4	127.1 (4)
C5—C6—C7	119.0 (3)	O16—C28—H28	116.5
C5—C6—C2	121.8 (3)	N4—C28—H28	116.5
C7—C6—C2	118.9 (3)	N4—C29—H29A	109.5
C8—C7—C6	120.7 (3)	N4—C29—H29B	109.5
C8—C7—H7A	119.6	H29A—C29—H29B	109.5
C6—C7—H7A	119.6	N4—C29—H29C	109.5
C9—C8—C7	119.5 (3)	H29A—C29—H29C	109.5
C9—C8—C3	119.5 (3)	H29B—C29—H29C	109.5
C7—C8—C3	120.9 (3)	N4—C30—H30A	109.5
C8—C9—C4	120.6 (3)	N4—C30—H30B	109.5
C8—C9—H9A	119.7	H30A—C30—H30B	109.5
C4—C9—H9A	119.7	N4—C30—H30C	109.5
O7—C10—O8	125.4 (3)	H30A—C30—H30C	109.5
O7—C10—C13	119.5 (3)	H30B—C30—H30C	109.5
			
O1^i^—Mn1—O1—C1	−5 (100)	O6—C3—C8—C9	−172.0 (3)
O7^i^—Mn1—O1—C1	−109.8 (3)	O5—C3—C8—C7	−169.0 (3)
O7—Mn1—O1—C1	70.2 (3)	O6—C3—C8—C7	10.8 (5)
O13^i^—Mn1—O1—C1	157.6 (3)	C7—C8—C9—C4	−2.0 (5)
O13—Mn1—O1—C1	−22.4 (3)	C3—C8—C9—C4	−179.3 (3)
O1^i^—Mn1—O7—C10	116.8 (3)	C5—C4—C9—C8	2.2 (5)
O1—Mn1—O7—C10	−63.2 (3)	C1—C4—C9—C8	178.0 (3)
O7^i^—Mn1—O7—C10	49 (100)	Mn1—O7—C10—O8	24.4 (5)
O13^i^—Mn1—O7—C10	−148.6 (3)	Mn1—O7—C10—C13	−156.3 (2)
O13—Mn1—O7—C10	31.4 (3)	Mn2—O10—C11—O9	42.7 (5)
O12^ii^—Mn2—O10—C11	117.4 (3)	Mn2—O10—C11—C15	−136.3 (3)
O17—Mn2—O10—C11	122.5 (5)	Mn2^ii^—O12—C12—O11	45.8 (5)
O5^iii^—Mn2—O10—C11	−66.3 (3)	Mn2^ii^—O12—C12—C17	−135.1 (2)
O4^i^—Mn2—O10—C11	19.9 (3)	O7—C10—C13—C18	−169.7 (3)
O14—Mn2—O10—C11	−151.6 (3)	O8—C10—C13—C18	9.7 (5)
O1^i^—Mn1—O13—C19	47.3 (3)	O7—C10—C13—C14	11.2 (5)
O1—Mn1—O13—C19	−132.7 (3)	O8—C10—C13—C14	−169.4 (3)
O7^i^—Mn1—O13—C19	−46.2 (3)	C18—C13—C14—C15	−2.2 (5)
O7—Mn1—O13—C19	133.8 (3)	C10—C13—C14—C15	176.9 (3)
O13^i^—Mn1—O13—C19	76 (100)	C13—C14—C15—C16	2.3 (5)
O12^ii^—Mn2—O14—C22	−55.8 (3)	C13—C14—C15—C11	−173.6 (3)
O17—Mn2—O14—C22	29.6 (3)	O10—C11—C15—C14	158.2 (3)
O5^iii^—Mn2—O14—C22	121.1 (3)	O9—C11—C15—C14	−20.9 (5)
O10—Mn2—O14—C22	−140.1 (3)	O10—C11—C15—C16	−17.7 (5)
O4^i^—Mn2—O14—C22	145.4 (5)	O9—C11—C15—C16	163.2 (3)
Mn1—O1—C1—O2	−37.4 (5)	C14—C15—C16—C17	−0.1 (5)
Mn1—O1—C1—C4	142.3 (3)	C11—C15—C16—C17	175.8 (3)
Mn2^i^—O4—C2—O3	−42.0 (5)	C15—C16—C17—C18	−2.2 (5)
Mn2^i^—O4—C2—C6	135.3 (3)	C15—C16—C17—C12	−179.9 (3)
Mn2^iv^—O5—C3—O6	−25.9 (6)	O12—C12—C17—C16	176.9 (3)
Mn2^iv^—O5—C3—C8	154.0 (2)	O11—C12—C17—C16	−3.9 (5)
O1—C1—C4—C5	−2.6 (5)	O12—C12—C17—C18	−0.8 (5)
O2—C1—C4—C5	177.0 (3)	O11—C12—C17—C18	178.4 (3)
O1—C1—C4—C9	−178.4 (3)	C14—C13—C18—C17	−0.2 (5)
O2—C1—C4—C9	1.2 (5)	C10—C13—C18—C17	−179.3 (3)
C9—C4—C5—C6	−0.7 (5)	C16—C17—C18—C13	2.3 (5)
C1—C4—C5—C6	−176.6 (3)	C12—C17—C18—C13	−179.9 (3)
C4—C5—C6—C7	−0.9 (5)	Mn1—O13—C19—N1	−171.1 (3)
C4—C5—C6—C2	172.7 (3)	C21—N1—C19—O13	0.9 (7)
O4—C2—C6—C5	−166.6 (3)	C20—N1—C19—O13	−177.4 (5)
O3—C2—C6—C5	11.0 (5)	Mn2—O14—C22—N2	−171.6 (3)
O4—C2—C6—C7	7.1 (5)	C23—N2—C22—O14	−0.8 (8)
O3—C2—C6—C7	−175.4 (3)	C24—N2—C22—O14	179.8 (5)
C5—C6—C7—C8	1.1 (5)	C27—N3—C25—O15	−2.2 (7)
C2—C6—C7—C8	−172.8 (3)	C26—N3—C25—O15	179.2 (4)
C6—C7—C8—C9	0.4 (5)	C30—N4—C28—O16	−1.1 (7)
C6—C7—C8—C3	177.6 (3)	C29—N4—C28—O16	−179.9 (4)
O5—C3—C8—C9	8.2 (5)		

Symmetry codes: (i) −*x*+2, −*y*, −*z*+2; (ii) −*x*+1, −*y*+1, −*z*+2; (iii) *x*, *y*+1, *z*−1; (iv) *x*, *y*−1, *z*+1.

### Hydrogen-bond geometry (Å, º)

**Table:**

*D*—H···*A*	*D*—H	H···*A*	*D*···*A*	*D*—H···*A*
O8—H8···O2	0.82	1.67	2.448 (3)	157
O17—H2*W*···O16	0.85	1.85	2.694 (4)	176
O6—H6···O9^iv^	0.82	1.69	2.452 (3)	153
O3—H3···O11^v^	0.82	1.69	2.463 (3)	157
O17—H1*W*···O15^vi^	0.85	1.92	2.713 (4)	155

Symmetry codes: (iv) *x*, *y*−1, *z*+1; (v) *x*+1, *y*−1, *z*; (vi) −*x*+1, −*y*+1, −*z*+1.
